# Finding individualised treatment in obese needing enoxaparin (FIT ONE): a multicentre study of therapeutic enoxaparin and the role of anti-factor Xa monitoring

**DOI:** 10.1007/s11239-024-03033-7

**Published:** 2024-08-27

**Authors:** Marcelle Appay, Justine Lai, Justine Hay, Connie Calvisi, Geoffrey Wills, Shreyas Kharadi, Sajani Nanayakkara, Ji Sang Ryu, Rozanna Alameddine, Sarah Jupp, Margaretta Lin, Jessica Nguyen, Tammy Nguyen, Nicholas Harrison, Fady Gad, Sakura Kagaya, Liam Nguyen, Sharma Piyush, Vicky Shion, Advait Pandya, Mustafa Emin, Ewe Shen Lim, Urna Rahman, Farhad Hayat, Chamali Gajaweera, Nashwa Sheriff, Asad E. Patanwala, Leonardo Pasalic, Jan-Willem Alffenaar

**Affiliations:** 1 School of Pharmacy, Faculty of Medicine and Health, University of Sydney, Science Rd, Camperdown, NSW 2050 Australia; 2 Department of Pharmacy, John Hunter Hospital, Lookout Rd, New Lambton Heights, NSW 2305 Australia; 3 Department of Pharmacy, Westmead Hospital, Cnr Hawkesbury Rd and Darcy Rd, Westmead, NSW 2145 Australia; 4 Department of Pharmacy, Nepean Hospital, Somerset St, Kingswood, NSW 2747 Australia; 5 Department of Pharmacy, Bankstown-Lidcombe Hospital, Eldridge Rd, Bankstown, NSW 2200 Australia; 6 Department of Pharmacy, Blacktown-Mount Druitt Hospital, Blacktown Rd, Blacktown, NSW 2148 Australia; 7 Department of Pharmacy, Royal Prince Alfred Hospital, Missenden Rd, Camperdown, NSW 2050 Australia; 8 Department of Pharmacy, Auburn Hospital, Hargrave Rd, Auburn, NSW 2144 Australia; 9 Medical Service, Blacktown-Mount Druitt Hospital, Blacktown Rd, Blacktown, NSW 2418 Australia; 10 School of Medicine, Faculty of Medicine and Health, University of Sydney, Science Rd, Camperdown, NSW 2050 Australia; 11 Institute of Clinical Pathology and Research (ICPMR), NSW Health Pathology, Hawkesbury Rd, Westmead, NSW 2145 Australia; 12 Department of Haematology, Westmead Hospital, Cnr Hawkesbury Rd and Darcy Rd, Westmead, NSW 2145 Australia

**Keywords:** Enoxaparin, Treatment, Therapeutic, Obese, Obesity, LMWH, DVT, PE

## Abstract

Enoxaparin is dosed according to actual body weight in treatment of arterial and venous thrombosis. Due to its hydrophilic nature, it distributes according to lean body mass which may be problematic when dosing obese patients as this may increase the risk of bleeding events in this population. The aim was to evaluate current therapeutic enoxaparin dosing strategies, including Antifactor Xa (AFXa) level monitoring, in obese patients and to identify factors that contribute to treatment failure and excess anticoagulation. A retrospective cohort study was conducted reviewing patients administered therapeutic enoxaparin between May 2020 and April 2021. Data were collected on patient characteristics, enoxaparin therapy, AFXa monitoring, and outcomes. Regression models were constructed to assess variables of interest to estimate any association with AFXa levels. In total 762 patients were included in the analysis. The mean initial weight-based dose was 0.95 mg/kg twice daily (SD: ± 0.12, IQR 0.92–1.01) and 1.04 mg/kg once daily (SD: ± 0.26, IQR 0.93–1.12) and 14.4% of patients had AFXa monitoring. Treatment failure was experienced by 2.2%, 5% experienced bleeding. There was no association between the mean actual milligram per kilogram weight-based twice daily doses and subtherapeutic, therapeutic and supratherapeutic AFXa levels (P = 0.135). Obesity was not included in the final regression models due to lack of significance. At a mean therapeutic enoxaparin dose of 0.95 mg/kg twice daily and 1.04 mg/kg once daily no excess in treatment failure or bleeding events were observed in obese patients compared to the product information. Obesity was not an independent variable that affected the achievement of target AFXa levels.

**Graphical abstract:**

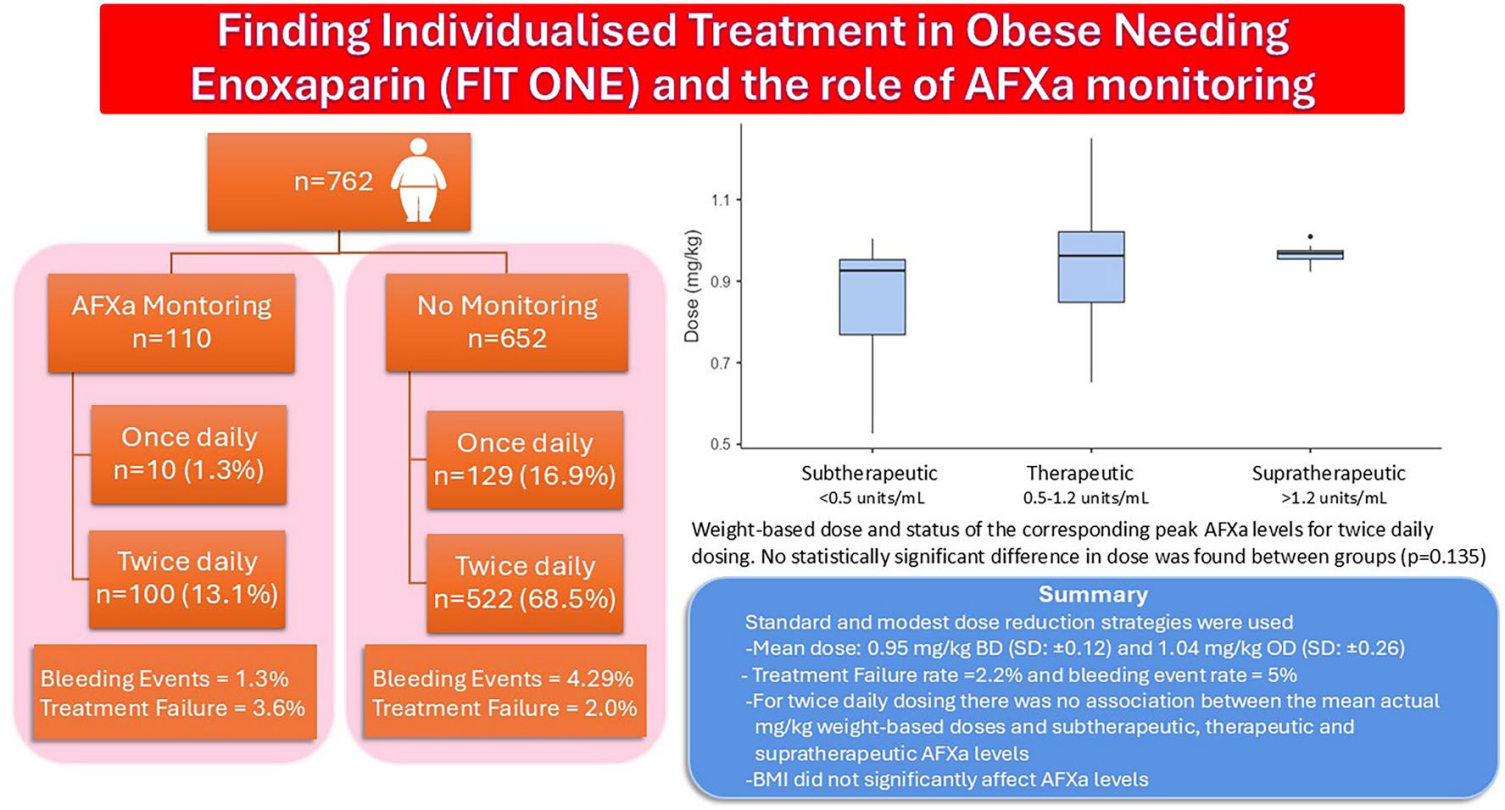

## Highlights

- A large multisite study showing that standard weight-based dosing and moderately reduced dosing strategies may not increase the incidence of treatment failure and may not increase the risk of bleeding in the obese population.
- Degree of obesity, as measured by BMI, BMI class or weight, was not an independent variable that affects the achievement of target therapeutic AFXa levels.
- There was no association between the mean actual milligram per kilogram weight-based twice daily doses and subtherapeutic, therapeutic and supratherapeutic AFXa levels (P = 0.135)

## Introduction

Enoxaparin is a low molecular weight heparin (LMWH) used to treat both arterial and venous thrombosis. It exerts its antithrombotic effect through actions on the coagulation pathway primarily through the inhibition of two main coagulation factors: Factor Xa and thrombin [1]. Due to its hydrophilicity, enoxaparin concentrates in extracellular fluid, blood and lean tissue and does not distribute into adipose tissue [1]. For treatment indications, it is conventionally dosed according to actual body weight at 1 mg/kg twice a day or 1.5 mg/kg once daily with no maximum dose listed in product information [1]. This may pose an issue when dosing obese patients due to their higher ratios of adipose tissue to lean tissue as well as physiological changes that alter drug pharmacokinetics caused by hormone dysregulation and cytokine secretion in fat tissue [2–5]. Therefore, weight-based dosing using actual body weight may increase the antithrombotic activity of enoxaparin and increase the risk of bleeding events in this population [6].

Globally, according to the World Health Organisation (WHO), obesity, defined as a body mass index (BMI) of ≥ 30 kg/m^2^, has now reached epidemic proportions [7]. Australian data collected in 2022 demonstrates that 32% of adults are obese with 13% further classified as living with severe obesity (BMI ≥ 35 kg/m^2^) [8]. Additionally, obesity causes a hypercoagulable state, impairs fibrinolysis and causes chronic inflammation making it a strong independent risk factor for thrombosis [9]. Hence, effective treatment of this sub-population is important due to its high prevalence and higher risk of disease. Further complicating dosing, obese patients are often excluded from or underrepresented in pharmacokinetic and clinical trials, thus evidence supporting dosing recommendations in this population is limited [10, 11]. Due to its predictable ability to inhibit factor Xa, the level of anticoagulation in patients treated with enoxaparin can be assessed by using antifactor Xa (AFXa) levels as a surrogate marker. This often occurs in clinical practice for certain high-risk patient populations including those with obesity, however target ranges have not been clinically validated and have been defined by consensus of experts [12]. The European Society for Vascular Surgery, the American Society of Hematology and the Thrombosis and Haemostasis Society of Australia and New Zealand currently do not recommend or do not provide any guidance regarding AFXa monitoring in the obese [13–16]. Where a recommendation is provided the guidelines state it is based on very low certainty in the evidence about effect. Additionally, these guidelines only recommend weight-based dosing using total body weight but do not provide specific guidance on dose modification or monitoring in obese populations [13–15]. Additionally, there is limited research examining dosing of therapeutic enoxaparin in obesity with the majority of studies being underpowered, single centre studies with small sample sizes. A recent review on this topic found that current studies are inadequate to determine an ideal dosing strategy and that individualising doses according to AFXa levels may be appropriate to guide dose adjustment in this population [17].

The aim of this study was to evaluate current therapeutic enoxaparin weight-based dosing strategies, and the use of AFXa level monitoring to individualise treatment in obese patients in a large multicentre cohort study. Secondary objectives were to identify factors that may contribute to enoxaparin treatment failure and excess anticoagulation.

## Method

### Study design and setting

This retrospective study was conducted at 7 principal referral and public acute hospitals in New South Wales, Australia, evaluating all obese patients who were prescribed therapeutic enoxaparin between May 2020 and April 2021 [18]. AFXa levels were collected at the discretion of attending physicians and were part of routine care. The hospitals were Auburn Hospital, Bankstown-Lidcombe Hospital, Blacktown-Mount Druitt Hospital, John Hunter Hospital, Nepean Hospital, Royal Prince Alfred, and Westmead Hospital.

### Ethics

The study was approved by the Hunter New England Human Research Ethics Committee (2021/ETH01300). Due to the retrospective nature of this study a waiver of patient informed consent was granted.

### Patients

Patients were eligible for inclusion in this study if they were ≥ 18 years old and had a body mass index (BMI) ≥ 30 kg/m^2^ or a weight ≥ 120 kg. Using the electronic prescribing system at each facility, a list of all adult patients who had a new order for enoxaparin during their admission who met the weight or BMI criteria was generated. Patients were excluded from the study for the following reasons: (1) enoxaparin was not prescribed for a therapeutic indication, (2) pregnancy, or (3) dialysis. Atrial fibrillation was excluded as a therapeutic indication despite the fact it is usually dosed at therapeutic doses as it is considered a prophylactic indication, and pregnant and dialysed patients were excluded due to altered pharmacokinetics. When patients were administered multiple courses of therapeutic enoxaparin during the study period, only the first course that met the inclusion criteria was included. The end of a course of treatment was defined as the electronic order being ceased for 24 h or more rather than doses being withheld in the system.

### Data collection

Relevant data were extracted from patient’s electronic medical records, deidentified and recorded into REDCap 13.4.10. Data collected included patient characteristics (weight, height, BMI, age, sex, comorbidities, medicines that affect bleeding and baseline serum creatinine), haematology involvement, patient hospital location, enoxaparin therapy (dose, frequency, duration and indication), bleeding and thrombotic risk, AFXa monitoring and outcomes (formation of new thrombus, progression of existing thrombus, myocardial infarction, stroke and bleeding). Outcome data was collected from the duration of the admission and if the patient was subsequently re-admitted within 30 days of discharge. Events that happened in the outpatient setting not resulting in admission could not be captured.

### Measurement of anti-factor Xa level

Anti-factor Xa levels were measured by 3 National Association of Testing Authorities (NATA)-accredited laboratories which are part of a standardised network of laboratories comprising the New South Wales Health Pathology [19]. Blood samples were collected in sodium citrate tubes, which were double centrifuged, and the plasma removed. At facilities where the laboratory was onsite the sample was sent directly to the local laboratory. In line with quality standards, where the testing laboratory was off-site the sample was frozen for transport [19, 20]. The HemosIL Liquid Anti-Xa kit was used to determine the concentration of Low Molecular Weight Heparin (LMWH). The assay is a one stage chromogenic assay based on a synthetic chromogenic substrate and Factor Xa. The linear range for this test is 0.05–2.0 U/mL [19].

### Definitions

BMI was defined as a patient’s weight in kilograms divided by the square of their height in meters [7]. Obesity was defined as a BMI of greater than 30 kg/m^2^ and it was further classified by class: BMI of ≥ 30– < 35 kg/m^2^ was class 1, ≥ 35– < 40 kg/m^2^ was class 2 and ≥ 40 kg/m^2^ was class 3 [21].

Correctly taken peak concentrations were defined as samples that were taken within 3–5 h of the last documented enoxaparin dose given once steady state concentration was achieved [1]. In line with product information, steady state was defined as after the third treatment dose. The therapeutic range was defined as 0.5–1.2 units/mL for twelve hourly dosing and 1–2 units/mL for once daily dosing [19]. In cases where multiple AFXa levels were collected in the same patient only the first steady state level recorded was used when assessing the prescribed dose in relation to achieving the therapeutic range.

Treatment failure was defined as clinically confirmed development or progression of either venous or arterial thrombotic events. Adverse bleeding events that occurred during enoxaparin treatment up to 24 h after cessation were captured and further categorised according to the International Society of Thrombosis and Haemostasis criteria for major and minor bleeding [22, 23].

Padua prediction score was used to assess venous thromboembolism (VTE) risk for each patient at baseline and the HAS-BLED tool was used to assess bleeding risk with higher scores indicating greater risk for both scales [24, 25].

### Statistical analysis

Descriptive statistics were conducted for all patients. Due to the retrospective, noninterventional design of the study and the predicted low frequency of treatment failure and major bleeding events, no power calculation was performed. A P-value of < 0.05 was considered significant. Statistical comparison between patients who received AFXa monitoring and those who did not were performed using the independent student t test for continuous parametric variables, the Mann–Whitney U test for continuous, non-parametric variables and the χ^2^ test was used for categorical variables. Welch’s ANOVA test was used to compare the weight-based doses that resulted in subtherapeutic, therapeutic and supratherapeutic peak AFXa levels. In the subset of patients who had a correctly taken peak AFXa level, univariate and multivariable linear regression models were constructed to assess variables of interest to estimate any association with AFXa levels. The forward stepwise approach was used to assess the following variables in the linear models: age, sex, BMI, baseline serum creatinine, and weight-based enoxaparin dose (mg/kg) at the time the AFXa level was taken. The correlation between pairs of the numeric predictor variables considered for inclusion in the multivariable model were calculated to assess the model assumption of collinearity. High correlation was considered to be r > 0.7. Univariate and multivariable logistic regression models were used to assess any independent risk factors that were associated with treatment failure and bleeding events. The forward stepwise approach was used to assess the following variables in the logistic models: age, sex, BMI, baseline serum creatinine, Padua prediction score, HAS-BLED score and initial weight-based enoxaparin total daily dose (mg/kg/day). Univariate factors with a P value < 0.25 were tested in the final models.

## Results

### Patient population

**Fig. 1:**
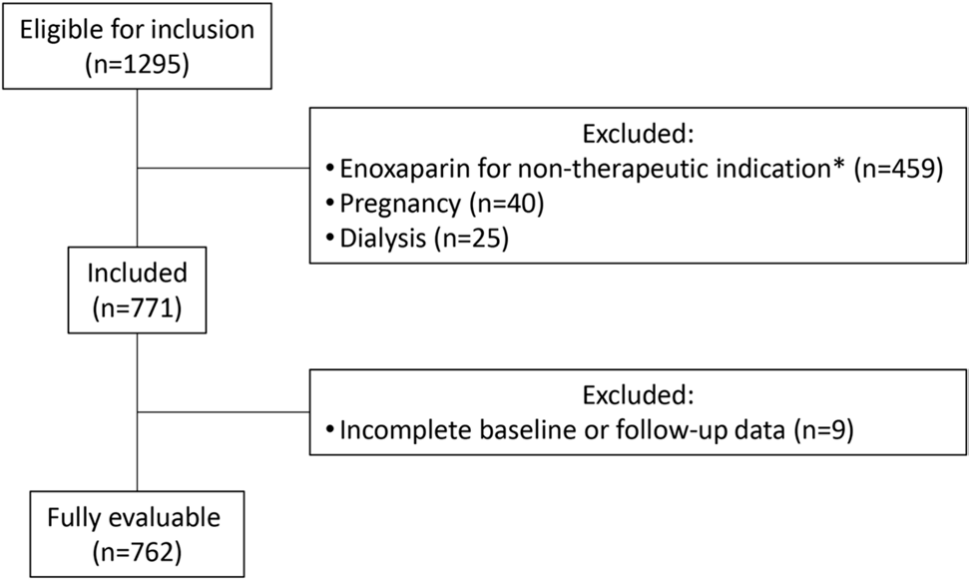
Patient Inclusion Flow Diagram. *Non-therapeutic indications included: anticoagulant bridging prior to surgery, warfarin bridging for atrial fibrillation and prophylaxis

During the study period 1295 obese patients were prescribed enoxaparin at therapeutic doses. Patients were excluded from the study if they had one or more of the pre-determined exclusion criteria. A total of 533 patients were excluded; 459 were prescribed enoxaparin for a non-therapeutic indication, 40 were pregnant and 25 were on dialysis. A further 9 patients were excluded due to incomplete ‘baseline’ or ‘enoxaparin therapy’ data. This resulted in 762 patients included in the final analysis (Fig. 1). The demographics of the final patient cohort are detailed in Table 1. The cohort was 52% male (n = 398), and the mean age was 61.8 years (SD: ± 15.1, range: 18–99). The median weight was 100 kg (IQR 89–115 kg) with the heaviest patient weighing 263 kg, and the median BMI was 35 kg/m^2^ (IQR 32.3–39.4 kg/m^2^), range: 30–82.5 kg/m^2^) (see Table 1).

**Table 1:** Patient characteristics (n = 762)

	All (n = 762)	With AFXa level (n = 110)	Without AFXa level (n = 652)		P value
Male	396 (52)	50 (45.5)	346 (53)		0.136
Age, years, mean (SD)	61.8 (± 15.1)	57.9 (± 14.9)	62.5 (± 15.1)		0.003**
Weight, kg, median (IQR)	100 (89.0–115.0)	111.5 (94.2–130.0)	100 (88.2–112.0)		< 0.001**
BMI^a^, kg/m^2^, median (IQR)	35 (32.3–39.4)	37.9 (33.2–44.9)	34.7 (32.2–38.8)		< 0.001**
BMI class^a^					< 0.001**
Class 1 (≥ 30–< 35 kg/m^2^)	378 (50.0)	37 (33.6)	341 (52.3)		
Class 2 (≥ 35–< 40 kg/m^2^)	204 (27.0)	26 (23.6)	178 (27.3)		
Class 3 (≥ 40 kg/m^2^)	174 (23.0)	45 (40.9)	129 (19.7)		
Baseline serum creatinine, micromol/L, median (IQR)	78 (64.3–97.0)	76 (61.3–100.0)	78 (65.0–96.0)		0.366
Initial enoxaparin dose					
Total daily dose, mg, mean (SD)	182.3 (± 52.2)	200.5 (± 55.2)	179.2 (± 51.0)		< 0.001**
Actual weight based dose once daily, mg/kg, mean, SD	1.04 ± 0.26	1.00 ± 0.22	1.05 ± 0.26		
Actual weight based dose twice daily, mg/kg, mean, SD	0.95 ± 0.12	0.91 ± 0.15	0.96 ± 0.43		
Actual weight based dose thrice daily, mg/kg, (n = 1)	0.98	0.98			
Duration of enoxaparin treatment^b,c^					
Total, days, median (IQR)	2.5 (1–5.5)	6.5 (4.5–12)	2.0 (1.0–4.5)		< 0.001**
Once daily, median (IQR)	2.0 (1.0–5)	5.25 (4.6–8.75)	2.0 (1.0–5.0)		
Twice daily, median (IQR)	3.0 (1.5–5.5)	7.0 (4.0–12.75)	2.5 (1–4.5)		
Thrice daily, mg/kg, (n = 1)	11.5		11.5		
Indication*					
PE	311 (40.8)	61 (55)	253 (38.6)		< 0.001**
DVT	220 (28.8)	39 (35.5)	181 (27.6)		0.094
NSTEMI	197 (25.8)	4 (3.6)	193 (26.5)		< 0.001**
Other^d^	105 (13.8)	22 (20)	25 (3.8)		
Padua prediction score, median (IQR)	4 (2–6)	5 (3–7)	4 (2–6)		< 0.001**
HAS-BLED score, median (IQR)	1 (1–2)	1 (0–2)	1 (1–2)		0.005**
Comorbidities					
Diabetes	249 (32.6)	35 (31.8)	214 (32.8)		0.836
Heart and/or respiratory failure	97 (12.7)	22 (20)	75 (11.5)		0.013**
Recent trauma/surgery (< 1 month)	76 (10.0)	24 (21.8)	52 (8.0)		< 0.001**
COPD	69 (9.1)	10 (9.1)	59 (9.0)		0.989
Thrombophilic conditions	51 (6.7)	11 (10)	40 (6.1)		0.133
Active cancer	122 (16.0)	25 (22.7)	97 (14.9)		0.038**
AF	44 (5.8)	3 (2.7)	41 (6.3)		0.139
Concomitant Medications^e^ that may increase risk of bleeding	405 (53.1)	44 (40)		361 (55.4)	0.003**

Data are presented as n (%) unless stated otherwise

*Percentages do not add up to 100% due to patients with more than 1 treatment indication

**P < 0.05

^a^6 patients did not have a calculated BMI as no height was recorded in the medical records

^b^Duration was rounded to the nearest half day

^c^4 patients did not have a calculated duration as the end date of treatment was not recorded

^d^Other indications: Portal Vein Thrombosis, Cerebral Venous Sinus Thrombosis, Upper Extremity DVT, Ischaemic Stroke, Renal infarct, Renal Vein Thrombosis, Splenic Infarct, Splenic Vein Thrombosis, Left Atrial Thrombus, Left Ventricular Thrombus, STEMI, Unstable Angina, Left Atrial Appendage Thrombus, Aortic Thrombus, Superficial Thrombophlebitis, Superficial Venous Thrombosis, Femoral Artery Stent Thrombosis, Mesenteric Vein Thrombosis, Mesenteric Artery Thrombosis, Lower Limb Arterial Thrombus, Critical Limb Ischaemia

^e^Concomitant medications: unfractionated heparin, warfarin, aspirin, clopidogrel, prasugrel, ticagrelor, eptifibatide, tirofiban, or non-steroidal anti-inflammatory drugs

### Treatment

The most common indications for treatment were pulmonary embolism (PE) (n = 314, 41%) followed by deep vein thrombosis (DVT) (n = 220, 28.8%) and non-ST-elevation myocardial infarction (NSTEMI) (n = 197, 25.8%). Patients prescribed enoxaparin for DVT and/or PE made up 62.2% (n = 474) of participants. The remaining indications accounted for less than 14% of patients and included thrombotic processes from the vascular, cardiac, neurological and portal vein systems. Some patients were prescribed enoxaparin for more than one indication.

### Enoxaparin dose

The mean initial total daily dose was 182.3 mg/day (S.D ± 52.2) and the median duration of treatment was 2.5 days (IQR 1–5.5). The majority of patients (n = 622) received twice daily dosing, 139 patients received once daily dosing, and 1 patient received thrice daily dosing with an initial total daily dose of 300 mg. The mean starting weight-based dose was 0.95 mg/kg (SD: ± 0.12, IQR 0.92–1.01) for twice daily dosing with a median duration of 3 days (IQR 1.5–5.5). A majority of patients (61.9%, n = 385) received a starting dose of less than 1 mg/kg twice daily with 19.6% (n = 122) of these patients prescribed a dose of less than 0.9 mg/kg. The mean initial weight-based dose was 1.04 mg/kg (SD: ± 0.26, IQR 0.93–1.12) for once daily dosing with a median duration of 2 days (IQR 1–5). 82.7% (n = 115) of once daily dosed patients receiving less than 90% of the recommended dose of 1.5 mg/kg. The single patient who was prescribed enoxaparin thrice daily was prescribed a dose of 0.98 mg/kg (See Table 1) for 11.5 days. Only 18.4% (n = 141) of patients had more than one dosing regimen prescribed with the majority of these patients, 84.4% (n = 119), having only one dose adjustment.

### Anti-factor Xa level monitoring

**Fig. 2:**
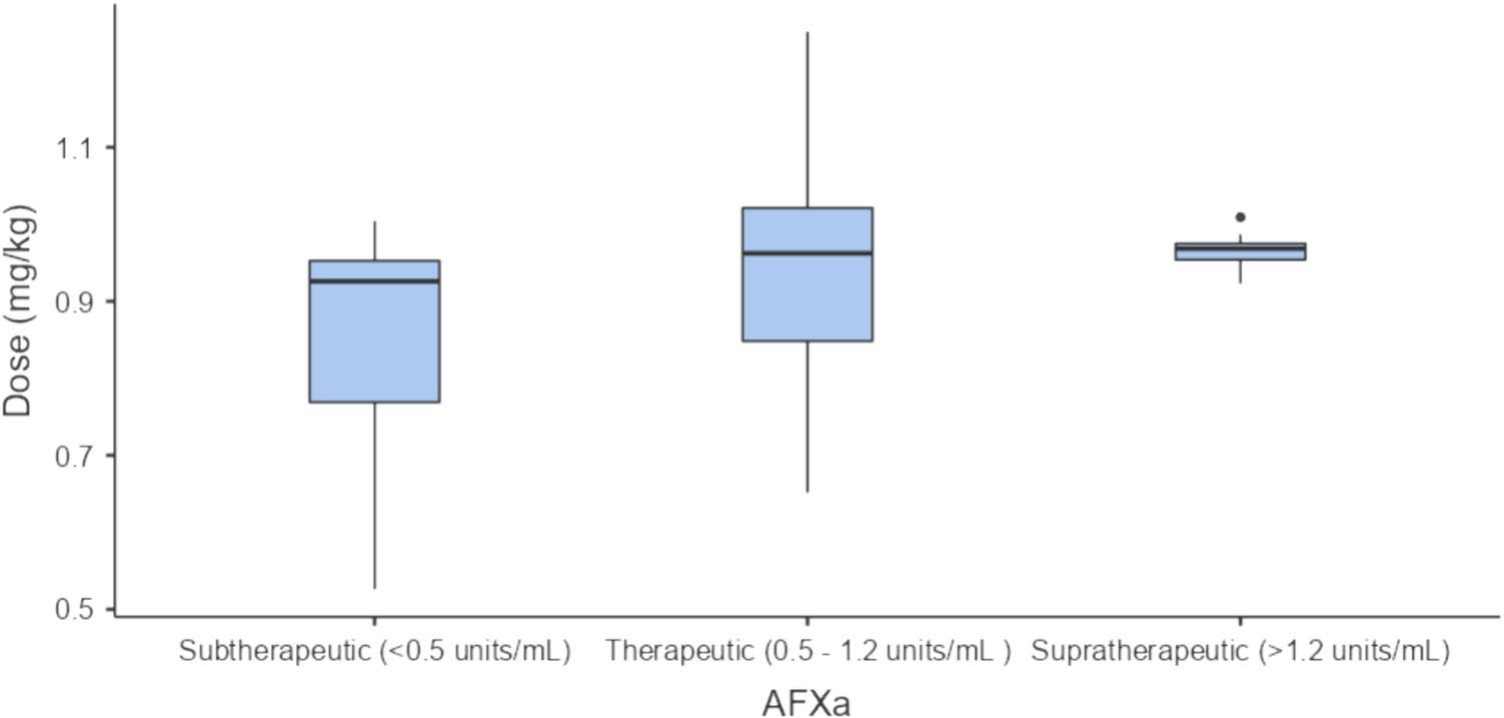
Weight-based enoxaparin dose and status of the corresponding first correctly taken peak AFXa levels (n = 45)—twice daily dosing. No statistically significant difference in dose was found between groups (P = 0.135)

Anti-Factor Xa (AFXa) level monitoring was measured for 110 patients (14.4%) with a total of 194 levels taken. Patients who had AFXa monitoring were significantly heavier than those who were not monitored with a median weight of 111.5 kg compared to 100 kg (P < 0.001) and a median BMI of 37.9 kg/m^2^ compared to 34.7 kg/m^2^ (P < 0.001) (see Table 1). The AFXa monitored group was also younger with a mean age of 57.9 years compared to 62.5 years for the unmonitored group (P = 0.003) and had a higher median Padua prediction score, median score of 5 for those who were monitored compared to a median score of 4 for the unmonitored group (P < 0.001). There was also evidence of an association between indication and monitoring with a higher proportion of the AFXa monitored group being treated for pulmonary embolism, 55% compared to 38.6% in the unmonitored group (P < 0.001) and a lower proportion being treated for NSTEMI, 3.6% of the monitored group compared to 26.5% in the unmonitored group (P < 0.001). Overall, 19.4% of PE patients, 17.7% of DVT patients and 2% of NSTEMI patients were monitored. Duration of treatment, patient hospital location and haematology involvement in care also affected monitoring. Patients who were monitored were prescribed enoxaparin for a significantly longer median duration with the monitored group having a median duration of 6.5 days compared to 2 days for the unmonitored group (P < 0.001). The percentage of patients treated with enoxaparin who received AFXa level monitoring varied greatly between sites with a median of 12.2% of patients receiving monitoring (range: 0–30.1%). Haematology involvement in care also affected monitoring with 74.5% of the AFXa monitored group receiving haematology involvement compared to 22.8% of those who were not monitored. Of the 194 levels that were taken only 60 peak levels (30.9%) from 48 patients were correctly taken within the 3–5 h after steady state was reached. The median time a level was taken after the last documented enoxaparin dose was given was 4 h and 44 min (range: 6 min–16 days). Of all correctly taken levels, 15.0% (n = 9) were subtherapeutic, 68.3% of levels (n = 41) were therapeutic and 16.7% (n = 10) were supratherapeutic. Prior to dose adjustment the median peak AFXa level was 0.88 units/mL (range: 0.29–1.77) and the median mg/kg dose was 0.96 mg/kg (IQR 0.85–1.01) for patients prescribed twice daily dosing (n = 45). The mean actual weight-based twice daily dose that was associated with subtherapeutic levels was 0.84 mg/kg (SD: ± 0.17), the mean actual weight-based dose that was associated with therapeutic levels was 0.93 mg/kg (SD: ± 0.13) and the mean dose that was associated with supratherapeutic levels was 0.96 mg/kg (SD: ± 0.03) (see Fig. 2). There was no significant difference between the mean twice daily doses for each group (P = 0.135). The BMI class of these patients was also assessed to determine if there was a difference between patients who had subtherapeutic, therapeutic or supratherapeutic levels however no statistically significant difference was found (P = 0.125).

The relationship between peak AFXa level and variables of interest for patients prescribed twice daily enoxaparin was examined using multivariable linear regression. To avoid collinearity, where a patient had multiple peak AFXa levels taken only the first recorded level was used (n = 45) and the other levels were excluded. After univariate analysis, the regression coefficients for the following variables met the requirement of a P value < 0.25: last weight-based enoxaparin dose given prior to the correctly taken peak AFXa level, baseline serum creatinine, age and sex. The regression coefficient for BMI was not statistically different from zero and was therefore not tested in the multivariable model (P = 0.688, see Table 2). The final multivariable model included the following variables: weight-based enoxaparin dose, baseline serum creatinine and sex (See Table 3). This model showed, after adjusting for baseline serum creatinine and sex, that an increase of 0.1 mg/kg in the weight-based dose increased AFXa level by 0.107 IU/mL (95% CI 0.034–0.181, P = 0.005). No high collinearity among the numeric predictor variables was detected (all r < 0.4).

**Table 2:** Univariate linear regression analysis of variables of interest for first peak correctly taken AFXa levels (n = 45)

Variable	Estimate	95% Confidence interval		T value	P value
		Lower	Upper		
Dose (mg/kg)	1.008	0.193	1.823	2.49	0.017
Baseline serum creatinine (micromol/L)	0.004	6.92 × 10^4^	0.008	2.40	0.021
Age (years)	0.007	3.73 × 10^6^	0.013	2.02	0.050
Sex (male–female)	− 0.182	− 0.399	0.035	− 1.69	0.098
BMI (kg/m^2^)	0.002	0.013	0.009	0.40	0.688

**Table 3:** Final multivariable linear regression model for first peak correctly taken AFXa levels (n = 45)

Variable	Estimate	95% confidence interval		T value	P value
		Lower	Upper		
Dose (mg/kg)	1.074	0.342	1.805	2.96	0.005
Baseline serum creatinine (micromol/L)	0.005	0.002	0.008	2.99	0.005
Sex (male–female)	− 0.196	− 0.383	− 0.008	2.102	0.042

### Factors associated with treatment failure

While being treated with enoxaparin 17 patients (2.2%) experienced treatment failure which resulted in death for three patients. There were 23 treatment failure events which included progression or development of PE (n = 11), DVT (n = 9), ischaemic stroke (n = 2) or myocardial infarction (n = 1). Only one patient had a correctly taken peak AFXa level within 24 h of treatment failure, this level was therapeutic (0.68 IU/mL). The majority of patients who experienced treatment failure were prescribed enoxaparin to initially treat either a PE (n = 8), a DVT (n = 1), or both (n = 6). This equates to a treatment failure rate of 3.1% (n = 15/474) in this group. Only these patients were included in the subsequent analysis. There were eight females and seven males in this group with a median BMI of 34.6 kg/m^2^ (range: 31.3–44) and a median weight of 103 kg (range: 80–135) (see Table 4). Patients were all prescribed enoxaparin with a frequency of twice a day and the median weight-based dose prescribed at the time of treatment failure was 1.0 mg/kg twice daily (IQR: 0.9–1.04). Due to small number of events, univariate logistic regression was used to assess any covariates that were associated with treatment failure in patients who were prescribed enoxaparin to treat PE and/or DVT. Only initial weight based daily dose (mg/kg/day), baseline serum creatinine (micromol/L), and Padua prediction score met the univariate variable requirement of a P value < 0.25 with only the Padua prediction score demonstrating statistically significantly correlation with treatment failure (P < 0.05). An increase of one point on the Padua prediction score increased the odds of treatment failure by 23% (Odds ratio = 1.23, 95% CI 1.04–1.45, P = 0.01, see Table 5).

**Table 4:** Treatment failure and bleeding events for patients prescribed enoxaparin for DVT/PE

	Treatment failure n (%)	Bleeding event n (%)
Total, n	15	26
Male	7 (45.5)	10 (38.5)
Age, years, median (range)	59 (31–87)	63.5 (23–92)
Weight, kg, median (range)	103 (80–135)	108.5 (70–183)
BMI^^^, kg/m^2^, median (range)	34.6 (31.3–44.1)	37.8 (30.3–59.8)
Baseline serum creatinine, micromol/L, median (range)	65 (32–119)	77.5 (32–135)
Padua prediction score, median (IQR)	5 (4–9)	7 (5.25–9)
HAS-BLED score, median (IQR)	1 (0–2)	1 (1–2)
Concomitant medications that may increase risk of bleeding	5 (33.3)	13 (50)

**Table 5:** Univariate logistic regression analysis of risk factors for treatment failure in patients treated for DVT and/or PE (n = 15)

Variable	Estimate	Odds ratio	95% CI	P value
Padua prediction score	0.209	1.23	1.05–1.45	0.012
Baseline serum creatinine (micromol/L)	− 0.017	0.98	0.96–1.01	0.138
Initial weight based daily dose (mg/kg/day)	0.902	2.46	0.55–10.98	0.237
BMI (kg/m^2^)	− 0.049	0.95	0.87–1.04	0.284
Age (years)	− 0.007	0.99	0.96–1.03	0.664
Sex (male–female)	0.134	1.14	0.41–3.21	0.799
HAS-BLED score	− 0.018	0.98	0.59–1.64	0.946

### Factors associated with bleeding

Thirty-eight patients (5%) experienced bleeding events while treated with enoxaparin with 31 events classified as minor and seven events classified as major. No events resulted in death. Only one patient had a correctly taken peak AFXa level within 24 h of the bleeding event, this level was therapeutic (0.68 IU/mL) and the bleeding event was classified as minor. More than half (68%, n = 26) of patients who experienced a bleeding event were initially prescribed enoxaparin to treat either a PE (n = 18), a DVT (n = 7), or both (n = 1). This equates to a bleeding rate of 5.5% (n = 26/474) for this patient group. The subsequent analysis was conducted for only patients prescribed enoxaparin for DVT and/or PE. There were 16 females and 10 males with a median BMI of 37.8 kg/m^2^ (range: 30.3–59.8) and a median weight of 108.5 kg (range: 70–183) (see Table 4). Twenty-four patients were prescribed twice daily dosing of enoxaparin while two were prescribed once daily dosing. The median weight-based dose of enoxaparin prescribed at the time of the bleeding event for twice daily dosing was 0.91 mg/kg (IQR 0.84–1.00) and for the two patients prescribed daily dosing was 1.0 mg/kg and 1.04 mg/kg. Univariate logistic regression was used to assess any independent risk factors that were associated with bleeding events in patients who were prescribed twice daily enoxaparin to treat PE and/or DVT. Only HAS-BLED Score and Padua prediction score met the univariate variable requirement of a P value < 0.25 with only the Padua prediction score demonstrating statistically significantly correlation with bleeding events (P < 0.05). An increase of one point on the Padua prediction score increased the odds of bleeding events by 31% (Odds ratio = 1.31, 95% CI 1.15–1.49, P < 0.001, see Table 6).

**Table 6:** Univariate logistic regression analysis of risk factors for bleeding events in patients treated for DVT and/or PE (n = 26)

Variable	Estimate	Odds ratio	95% CI	P value
Padua prediction score	0.269	1.31	1.15–1.49	< 0.001
HAS-BLED score	0.284	1.33	0.91–1.95	0.144
BMI (kg/m^2^)	0.019	1.02	0.98–1.06	0.345
Initial weight based daily dose (mg/kg/day)	0.442	1.56	0.56–4.35	0.399
Sex (male–female)	− 0.219	0.80	0.36–1.81	0.598
Age (years)	0.004	1.00	0.98–1.03	0.766
Baseline serum creatinine (micromol/L)	5.04 × 10^–4^	0.99	0.99–1.00	0.913

## Discussion

This large multicentre study investigating therapeutic dosing of enoxaparin in obese patients showed that 2.2% of patients experienced treatment failure while 5% experienced bleeding events. The patient cohort in this study was well represented with an even number of males and females, a wide range and even distribution of ages, and 50% of participants with a BMI > 35 kg/m^2^.

The majority of patients that were prescribed twice daily enoxaparin for the first dose were dosed in line with product information (mean 0.95 mg/kg ± 0.12) however 19.6% of patients were prescribed a dose less than 90% of the recommended dose of 1 mg/kg [1]. This finding is similar to previously published studies which reported a tendency for prescribers to initiate lower doses in obese patients [10, 26–28]. This practice was considerably more prevalent in our study in the group prescribed once daily dosing with 82.7% of patients receiving less than 90% of the recommended dose of 1.5 mg/kg (mean 1.04 mg/kg ± 0.26). This indicates that prescribers are less comfortable with prescribing obese patients with standard once daily doses. Despite this modest underdosing only 2.2% treatment failures were observed for all indications. The incidence of treatment failure in patients prescribed twice daily enoxaparin for DVT/PE was 3.1% and the median weight-based dose prescribed at the time of treatment failure was 1.0 mg/kg twice daily (IQR 0.9–1.04). This treatment failure rate is consistent with previously published studies which suggest that the true incidence in all patients prescribed twice daily therapeutic enoxaparin at recommended doses for DVT/PE lies somewhere between 3.1 and 5% [29]. This would therefore indicate that despite 19.6% of patients receiving starting doses at less than 90% of the recommended dose this does not seem to have increased the incidence of recurrence. This suggests that a modest dose reduction in obese patients may not compromise treatment efficacy. Furthermore, the univariate logistic regression analysis in our study did not show a link between BMI or actual weight based daily dose (mg/kg/day) and treatment failure. Instead, it demonstrated a positive association between Padua prediction score and treatment failure.

The incidence of bleed events in this study was also low at 5% for all patients and 5.5% for those treated for PE/DVT. This is lower than the incidence for PE/DVT patients reported in the product information which is 10% [1]. This can be explained by the median weight-based dose of enoxaparin prescribed at the time of the bleeding event for twice daily dosing which was almost 10% lower than recommended doses. Only the Padua prediction score demonstrated statistically significantly correlation with bleeding events (P < 0.05).

AFXa monitoring was performed in only 14.4% of patients which is a small proportion of patients included in the study. Only 60/194 AFXa levels were taken correctly which indicates that staff are not adequately aware of when and how to take levels. Patients who were monitored were on average 11.5 kg heavier and had higher BMIs. This suggests that prescribers were more likely to initiate monitoring in more obese patients. Indication and duration of therapy also appeared to be drivers for patient selection as those who were monitored were treated for longer durations and were more likely to be treated for pulmonary embolism. Patients treated for NSTEMI were far less likely to receive AFXa monitoring (P < 0.001), however, duration of treatment could have also influenced this result as NSTEMI patients usually receive short courses of enoxaparin [30]. Monitoring was also more likely to occur in patients where haematology was involved in care. It is worth noting that while the difference in Padua score and HAS-BLED score were also statistically significant between the two groups, this did not appear to have clinical significance as in both groups the median Padua score was greater than or equal to 4 which classifies a patient as high risk for VTE and the median HAS-BLED score was 1 for both groups which is considered moderate risk of bleeding [25]. Additionally, renal function, as measured by baseline serum creatinine, was not different between the two groups. This is an unexpected finding as international guidelines do recommend AFXa monitoring in patients with renal impairment and therefore we expected to see more patients with renal impairment in the monitored group [14]. However, as renal function was only collected for this study at the beginning of treatment the data may not capture patients who experienced significant renal decline while on enoxaparin who clinicians subsequently decided to monitor.

Due to the low number of patients who received monitoring at the time of their treatment failure or bleeding event our study was unable to link AFXa levels to outcomes. Only two patients had a correctly taken peak AFXa level at the time of the bleeding event or treatment failure and both levels were in the therapeutic range at the time of the event.

The results of this study found that in patients with a BMI ≥ 30 kg/m^2^ the actual BMI value was not a factor which influenced AFXa levels and BMI class did not affect whether a patient would have a subtherapeutic, therapeutic or supratherapeutic level. Instead AFXa levels were influenced by mg/kg dose based on actual body weight, baseline serum creatinine and gender. After adjusting for baseline serum creatinine and sex in the final multivariable linear regression model, an increase of 0.1 mg/kg in the weight-based dose increased AFXa levels by 0.107 IU/mL in obese patients. However, when examining the mean actual milligram per kilogram weight-based doses that resulted in subtherapeutic, therapeutic and supratherapeutic levels, our study found that there was no statistically significant difference between these doses. It can therefore be deduced that while a positive linear relationship exists between the minor differences in mg/kg dose based upon actual body weight these were too small to clinically impact on classification of AFXa levels as subtherapeutic or supratherapeutic in our study. This is likely due to the fact that the median dose prescribed when levels were taken was 0.96 mg/kg twice daily (IQR 0.85–1.01) which does not exceed the dose recommended in the product information. This suggests that doses within the range of 0.85–1 mg/kg twice daily are appropriate for this population. These results confirm findings of previous smaller studies (n = 26–54) [31–34]. Deal et al. which examined 26 morbidly obese patients with BMI > 40 kg/m^2^ found that there was no difference between the median doses patients with therapeutic levels and supratherapeutic levels received (0.74 mg/kg and 0.85 mg/kg respectively) [31]. Tahaineh et al. examined therapeutic dosing in 26 patients with a BMI > 30 kg/m^2^ and found that there was no statistically significant correlation between dose regimens (with and without capping) that were used and achieving therapeutic AFXa levels [32]. Finally Curry et al. a randomised controlled trial, comprising of 54 morbidly obese patients, compared standard dosing of 1 mg/kg twice daily to a reduced dose of 0.8 mg/kg twice daily and found that a similar proportion of patients in each group achieved therapeutic AFXa levels (89.3% vs. 76.9%) [33]. Our results do, however, conflict with the findings of Thompson-Moore et al. which examined 41 morbidly obese patients prescribed treatment enoxaparin. Similar to our study, Thompson-Moore et al. found a positive relationship between mg/kg dose and AFXa levels [6]. However, they found that mg/kg dosing based on actual body weight was an independent predictor of having a supratherapeutic AFXa level at steady state, OR 0.21 for < 0.95 mg/kg dosing versus ≥ 0.95 mg/kg dosing (95% CI 0.05–0.84). The dose of enoxaparin that resulted in therapeutic and supratherapeutic anti-Xa levels at steady state was significantly different, 0.83 mg/kg and 0.98 mg/kg respectively (− 0.11; 95% CI − 0.20 to − 0.01) [6].

One of the strengths of our study was the large, real world population sampled, which encompassed patients with a wide range of comorbidities, an even distribution of ages and BMIs and an even number of males and females. It examined a wide range of indications and dosing strategies and as a result it captured the current prescribing practices of clinicians. Therefore, the findings may be relevant to other settings with similar obese patient populations.

A limitation of this study was that, due to the retrospective nature, treatment failure and bleeding events relied upon review of patient clinical notes and pathology results. Treatment failure and bleeding events may not have been identified if documentation were incomplete and therefore there may have been more events than were identified and reported in the current results. A further limitation was the low number of AFXa levels taken during the study which limited the interpretation of these results. Additionally, the HAS-BLED and Padua prediction scores were calculated for study purposes and were not necessarily used by clinicians in all instances to guide decision making in real time.

## Conclusion

This large multicentred study found that physicians were more likely to monitor obese patients who had higher BMIs and those who were prescribed enoxaparin to treat pulmonary embolism. Those prescribed once daily frequencies were also more likely to be prescribed a reduced dose. The study found that standard weight-based dosing and moderately reduced dosing strategies may not increase the incidence of treatment failure and may not increase the risk of bleeding in the obese population. Additionally, the results indicate that degree of obesity, as measured by the actual BMI value was not a factor which influenced AFXa levels and that other factors, such as renal function, play a greater role. Additionally, BMI class was not found to affect whether a patient would have a subtherapeutic, therapeutic or supratherapeutic AFXa level.

## Data Availability

Data are available on request from the corresponding author.
